# PIWIL4 and SUPT5H combine to predict prognosis and immune landscape in intrahepatic cholangiocarcinoma

**DOI:** 10.1186/s12935-021-02310-2

**Published:** 2021-12-07

**Authors:** Wenbo Zou, Zizheng Wang, Xiuping Zhang, Shuai Xu, Fei Wang, Lincheng Li, Zhaoda Deng, Jing Wang, Ke Pan, Xinlan Ge, Chonghui Li, Rong Liu, Minggen Hu

**Affiliations:** 1 Medical School of Chinese PLA, Beijing, China; 2 Faculty of Hepato-Pancreato-Biliary Surgery, The First Medical Center of Chinese People’s Liberation Army (PLA) General Hospital, No.28 Fuxing Road, Haidian District, Beijing, 100853 China; 3 Institute of Hepatobiliary Surgery of Chinese PLA, Key Laboratory of Digital Hepetobiliary Surgery, PLA, Beijing, China; 4 School of Medicine, Nankai University, Tianjin, China

**Keywords:** Intrahepatic cholangiocarcinoma, Prognostic biomarkers, Bioinformatics, Targeted therapy

## Abstract

**Background:**

Intrahepatic cholangiocarcinoma (ICC) is a fatal primary liver cancer, and its long-term survival rate remains poor. RNA-binding proteins (RBPs) play an important role in critical cellular processes, failure of any one or more processes can lead to the development of multiple cancers. This study aimed to explore pivotal biomarkers and corresponding mechanisms to predict the prognosis of patients with ICC.

**Methods:**

The transcriptomic and clinical information of patients were collected from The Cancer Genome Atlas and Gene Expression Omnibus databases. Bioinformatic methods were used to identify survival-related and differentially-expressed biomarkers. Quantitative real-time PCR (qRT-PCR) and immunohistochemistry were used to detect the expression levels of key biomarkers in independent real-world cohorts. Subsequently, a prognostic signature was constructed that effectively distinguished patients in the high- and low-risk groups. Independent prognosis analysis was used to verify the signature’s independent predictive capabilities, and two nomograms were developed to predict survival.

**Results:**

PIWIL4 and SUPT5H were identified and considered as pivotal biomarkers, and the same expression trends of upregulation in ICC were also validated via qRT-PCR and immunohistochemistry in the separate real-world sample cohorts. The prognostic signature showed good predictive capabilities according to the area under the curve. The correlation of the biomarkers with the tumour microenvironment suggested that the high riskScore was positively related to the enrichment of resting natural killer cells and activated memory CD4 + T cells.

**Conclusion:**

In the present study, we demonstrated that PIWIL4 and SUPT5H could be used as novel prognostic biomarkers to develop a prognostic signature. This study provides potential biomarkers of prognostic value for patients with intrahepatic cholangiocarcinoma.

**Supplementary Information:**

The online version contains supplementary material available at 10.1186/s12935-021-02310-2.

## Background

Intrahepatic cholangiocarcinoma (ICC) is a fatal hepatobiliary malignancy derived from the bile ducts, and has a difficult diagnosis, poor prognosis, and very high chance of mortality [1, 2]. It accounts for approximately 10–20% of all bile duct malignancies [3]. To date, an increasing incidence of ICC has been reported in most of the world, while most patients with ICC have underlying liver diseases such as primary sclerotic cholangitis and hepatitis B, etc. [4, 5]. Surgery is considered the only treatment for cure at present, whereas most patients are not viable candidates for surgery at the time of diagnosis [6, 7]. Chemotherapy is another effective treatment, but most patients develop resistance eventually [8]. Therefore, finding novel and effective treatments to strengthen the long-term prognosis of ICC is necessary and urgent. Recently, immunotherapies and targeted therapies have gradually come to researchers' attention [9–12]. A variety of therapeutic targets have been identified and validated, and some vital biomarkers have been successfully transformed into clinical applications, such as FGFR, IDH1/2, PD1, PDL1 [11, 13–15]. However, there are still many undiscovered biomarkers, which play an important role in tumour progression; therefore, exploring additional prognostic biomarkers will help us better understand disease progression.

RNA-binding proteins (RBPs) play an important role in critical cellular processes, such as RNA splicing, modification, transport, localisation, stability, degradation, and translation [16]. These are essential for cell development, differentiation, and metabolism [17]. Thus, failure of any one or more of the above-mentioned processes can lead to the development of multiple diseases, including cancer [18–20]. Recent studies have demonstrated that RBP-mediated RNA modifications are critical for cancer progression via various molecular mechanisms, and that aberrant expression of RBPs in multiple cancer types affects the expression and function of oncogenes and anti-oncogenes [17, 21, 22].

Several studies have revealed that some crucial RBPs regulate tumorigenesis and cancer progression. For instance, the Musashi proteins, including Musashi-1 and Musashi-2, have been demonstrated as key prognostic biomarkers. Elevated Musashi protein expression characterises a variety of solid tumours and is associated with metastasis, lymph node invasion, and poor prognosis [23]. Increasing evidence suggests that the RBP HuR also plays a crucial role in disease progression by targeting the binding sites of oncogenes, or antioncogenes, and by regulating the corresponding cell processes [24, 25]. Similarly, elevated HuR expression is positively correlated with malignant biological behaviour in cancer. Notably, Toyota et al. demonstrated that high cytoplasmic HuR expression is closely associated with poor survival and the decreased efficacy of chemotherapy in patients with surgically resected cholangiocarcinoma [26]. Consequently, increasing evidence indicates that RBPs are extensively involved in tumour progression in ICC. At present, several RBPs and their underlying mechanisms have been revealed in the initiation and progression of cancer, while more promising targets for ICC need be identified and verified.

In the present study, we identified and validated prognosis-related biomarkers PIWIL4 and SUPT5H using bioinformatic methods based on multiple public databases and as a result developed a risk classification model to predict the long-term survival of ICC patients. Notably, the biomarkers were further verified in ICC and normal tissues via laboratory experiments, which showed a similar expression trend to each other.

## Materials and methods

### Data collection

The RNA sequences regarding cholangiocarcinoma, specifically TCGA-CHOL, and clinicopathological information were obtained from The Cancer Genome Atlas (https://portal.gdc.cancer.gov/) and allocated into the training cohort. The GSE107943 validation cohort’s transcriptome and clinicopathological data was collected from the Gene Expression Omnibus (GEO) database (https://www.ncbi.nlm.nih.gov/geo/). The list of 1542 RBPs was obtained based on a previous published study [27]. The mRNA matrix was annotated based on gene transfer format files from Ensemble using the Perl language. Tumour tissues with the pathological diagnosis of ICC and adjacent normal tissues were prospectively collected from the Chinese PLA General Hospital. Written informed consent was obtained from all patients. After surgical resection, all the tissues were immediately collected and stored at − 80 °C, awaiting further preparation for qRT-PCR analysis. The ICC tissue microarray for immunohistochemistry (IHC), which contained 155 ICCs and 5 adjacent normal tissues, was purchased from Shanghai Outdo Biotech Company and approved by the ethics committee of Shanghai Outdo Biotech Company (Shanghai, China).

### Extraction of RBP expression and differential expression analysis

Based on the obtained list of RBPs and mRNA expression profiles, we extracted the RBPs with available mRNA expression profiles from TCGA-CHOL and GSE107943 cohorts to construct a new matrix for subsequent analyses. Then, using the “limma” package [28], we screened the differentially expressed RBPs (DE-RBPs), with the thresholds set as false discovery rate (FDR) < 0.001 and |log2 fold-change (FC)|> 0.5. Gene Ontology (GO) and the Kyoto Encyclopaedia of Genes and Genomes (KEGG) enrichment analyses were used to annotate the upregulated and downregulated DE-RBPs via the “clusterProfiler” and “GOplot” packages [29, 30]. A protein–protein interaction network was constructed from the DE-RBPs using the STRING online database (https://string-db.org/) with a medium confidence threshold of 0.4, and was then visualised using Cytoscape software (version 3.7.2).

### Survival-related RBPs screening and copy-number variation (CNV) analysis

The univariate Cox regression analysis was implemented to find the survival-related DE-RBPs via the “survival” package, and the threshold was set to a p value < 0.05. Multivariate Cox regression analysis was used to determine the optimal survival-related DE-RBPs, and regression coefficients were noted for subsequent analysis. Kaplan–Meier (KM) curves were used to visualise the prognostic value of each DE-RBP in the TCGA cohort. To reveal the frequency of CNV of optimal DE-RBPs, we downloaded the CNV data of TCGA-CHOL from UCSC Xena (http://xena.ucsc.edu/), and revealed the RBPs location using the “RCircos” package [31].

### Quantitative real-time PCR

Total RNA extraction, cDNA synthesis, and qRT-PCR were performed according to the manufacturer’s protocol and our previous article [32]. h18S rDNA was used as an internal reference; finally, the cycle threshold (Ct) was recorded, and relative expression was calculated using the 2^−ΔΔCt^ method. Primer sequences of PIWIL4, SUPT5H, and h18S rDNA are shown in Table 1.

**Table 1:** Primers used for quantitative real-time PCR

GeneName	Direction	Sequences (5′–3′)
h18S	Forward	AACCCGTTGAACCCCATT
h18S	Reverse	CCATCCAATCGGTAGTAGCG
PIWIL4	Forward	CCAAGACTGGCAGCTATACCA
PIWIL4	Reverse	ACCGTCGAATGCTTTTGCTTT
SUPT5H	Forward	TGATCCCACGCATCGACTAC
SUPT5H	Reverse	TGGAGGCCGCTTAAACTTCTT

### Immunohistochemistry

All IHC staining for the tissue microarray was performed according to the manufacturer’s protocol. All images were obtained using a Leica Aperio XT digital pathology scanner (Leica, Wetzlar, Germany). The immunoreactive score (IRS) was calculated according to the ratio of positive cells and the staining intensity. This was used to evaluate expression levels between tumour and normal tissues. The ratio of positive cells was defined as follows: 0 (< 10%), 1 point (10–40%), 2 points (40–70%), and 3 (> 70%). The staining intensity was scored as 0 (negative), 1 (weakly positive), 2 (positive), and 3 (strongly positive). The two scores were added up to either 0–2 points to indicate weak expression, or 3–6 points, which was defined as strong expression. Detailed primary antibodies used for IHC are shown in Additional file 1: Table S1. The mean IRS from two random images of each sample was used to represent the final IRS using ImageJ software 1.53.

### Development and validation of RBP-related prognostic signature

By combining the coefficients and expression levels of PIWIL4 and SUPT5H, we generated the riskScore of each patient using the following formula: riskScore $= {\sum }_{i=1}^{k}\beta iexpi$. All patients were grouped into high- or low-risk groups based on the median riskScore. The KM survival curve presented the predictive power, and the area under the curve (AUC) of the receiver operating characteristic (ROC) curve was used to verify the prediction accuracy using the “survivalROC” package. We calculated the AUC at 1, 2, and 3 years to verify the model accuracy. Another independent gene expression profile, GSE107943, was used as the testing cohort for model validation. Finally, gene set enrichment analysis (GSEA) was performed to explore potential mechanisms, and principal component analysis was used to efficiently downscale high-dimensional sequencing data.

### Immune cell infiltration and chemotherapeutics efficacy analysis

We integrated acknowledged methods for evaluating the immune infiltration status in the TCGA-CHOL dataset, including TIMER [33], CIBERSORT [34], XCELL [35], QUANTISEQ [36], MCPcounter [37], and EPIC [38]. We analysed the correlation of the riskScore with immune cell infiltration [39], and used the TISIDB database (http://cis.hku.hk/TISIDB/) to explore the correlations between two RBPs expression and tumour immune microenvironment and immune-related markers.

To evaluate the capability of the signature to predict chemotherapeutic efficacy in ICC, we compared the half-inhibitory concentration (IC_50_) difference of several chemotherapeutics in high- and low-risk groups. The Wilcoxon signed-rank test and the “ggplot2” and the “pRRophetic” packages were used to implement this process [40].

### Optimal prognostic factors identification, clinical relevance analysis and nomogram construction

Univariate and multivariate regression analyses were performed to identify independent prognostic factors, including age, sex, tumour grade, American Joint Committee on Cancer (AJCC) stage, and peripheral nerve infiltration (PNI). Further survival analyses were also performed, stratified by clinical characteristics. Further, we generated two nomograms using the “rms” R package based on gene expression levels and clinicopathologic characteristics, respectively. The predictability of the nomograms was validated using the calibration curves.

### Statistical analysis

Continuous variables were reported as medians (interquartile range) and were analysed by the Student’s t-test, while a log-rank test was used to perform the survival analysis. All statistical analyses and graphics were performed using R version 4.0.2 and its resource packages. Overall survival (OS) was defined as the length of time between surgery and death, or the last follow-up. Two-sided p < 0.05, was considered statistically significant in all statistical tests.

## Results

### Data collection

In total, 1523 RBPs with available mRNA expression profiles were extracted for subsequent analyses. The gene symbols of all obtained RBPs are shown in Additional file 1: Table S2. A total of 33 patient samples were obtained from TCGA and allocated into the training cohort, while an additional 30 patient samples were collected from GEO and grouped into the testing cohort. The detailed survival information (OS or last follow-up time) and the clinicopathologic characteristics of the patients involved in the two cohorts are shown in Additional file 1: Table S3.

### Differential expression analysis

**Fig. 1:**
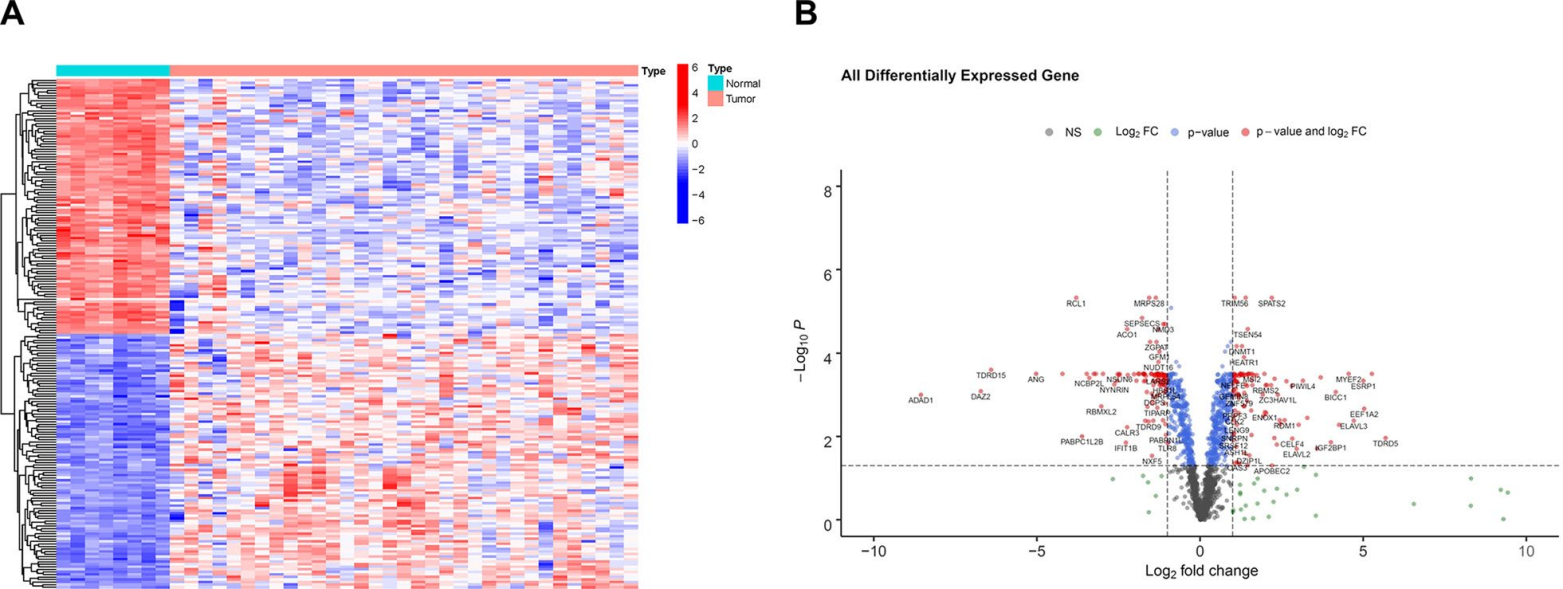
Differentially expressed analysis. **A** Heatmap of significant DE-RBPs. **B** Volcano plot of DE-RBPs

Using the screening criteria, a total of 242 DE-RBPs were identified, of which 116 were upregulated and 126 were downregulated (Fig. 1A, B, Additional file 1: Table S4). As expected, GO and the KEGG pathway analyses demonstrated that DE-RBPs were correlated significantly with vital RNA regulatory processes, such as RNA transport and RNA degradation (Additional file 2: Fig. S1A–D). The PPI network and corresponding subgroup revealed associations between these DE-RBPs (Additional file 2: Fig. S2A–D, Additional file 1: Table S5).

### Survival-related RBPs screening and CNV analysis

**Fig. 2:**
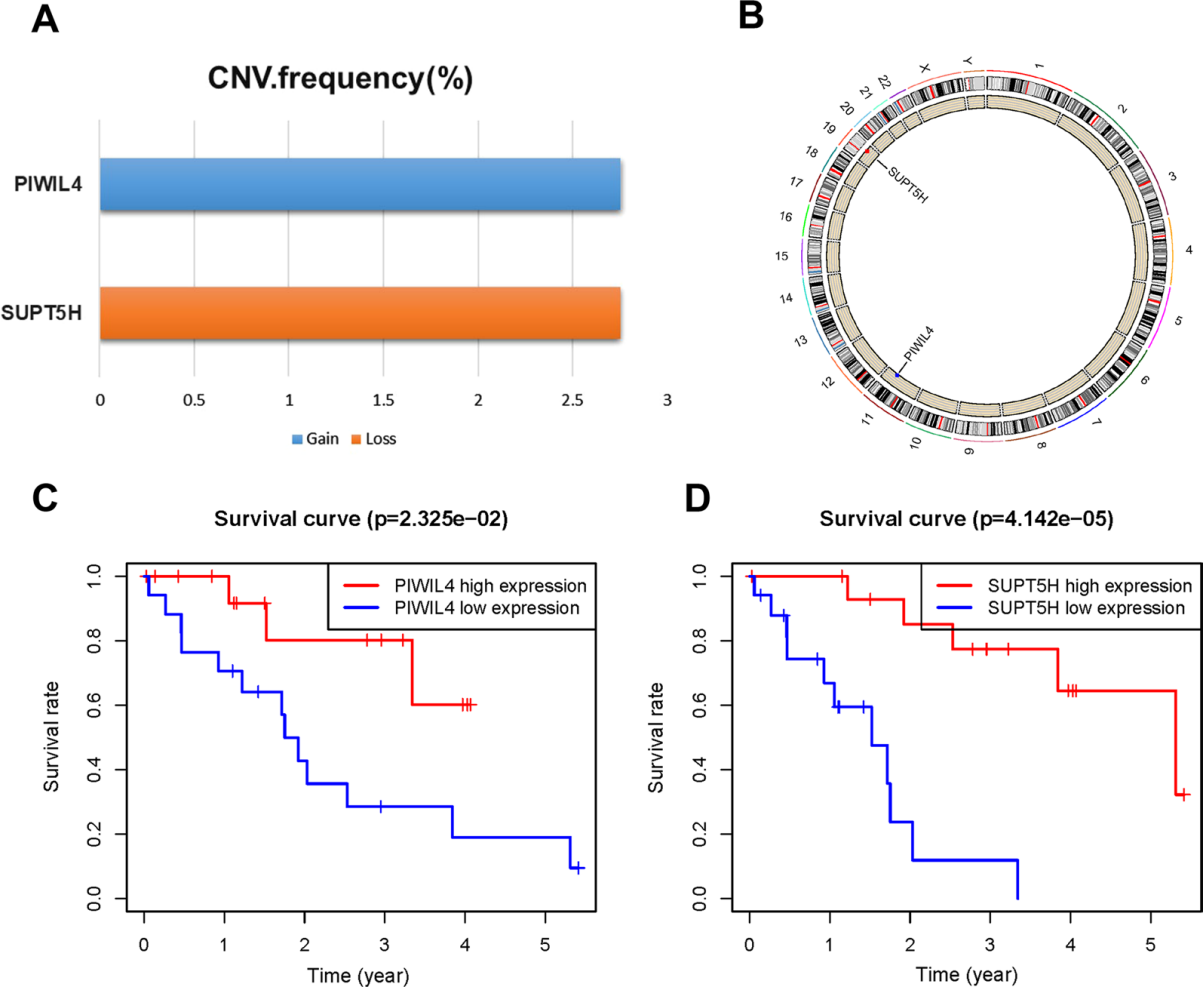
Copy number variation (CNV) analysis and KM survival analysis. **A** The barplot of CNV frequency (%). **B** The location of PIWIL4 and SUPT5H. **C** The KM survival curve of PIWIL4. D The KM survival curve of SUPT5H

Univariate and multivariate regression analyses identified two independently survival-related DE-RBPs, namely PIWIL4 and SUPT5H (Table 2), with the coefficients being less than 0, suggesting that they were key protective factors in tumour progression. Then, we revealed the CNVs of PIWIL4 and SUPT5H, which are shown in Fig. 2A and Additional file 1: Table S6. Notably, we also presented their location in the genome (Fig. 2B), which could help us understand the roles they might play. Finally, the KM survival curve indicated that both PIWIL4 and SUPT5H were protective factors for survival classification (p < 0.05, Fig. 2C, D).

**Table 2:** Univariate and multivariate Cox regression analysis for identifying independently prognostic biomarkers

Univariate Cox regression analysis				
Gene	HR	HR.95L	HR.95H	P value
PIWIL4	0.4006	0.1856	0.8648	0.0198
EIF4ENIF1	0.1630	0.0269	0.9895	0.0487
SUPT5H	0.0046	0.0003	0.0674	< 0.001
SCAF4	0.1656	0.0277	0.9894	0.0487

Multivariate Cox regression analysis					
Gene	Coef	HR	HR.95L	HR.95H	P value
PIWIL4	− 0.6601	0.5168	0.2138	1.2492	0.1427
SUPT5H	− 4.8675	0.0077	0.0005	0.1178	< 0.001

### Validation of expression of PIWIL4 and SUPT5H in the independent cohorts

**Fig. 3:**
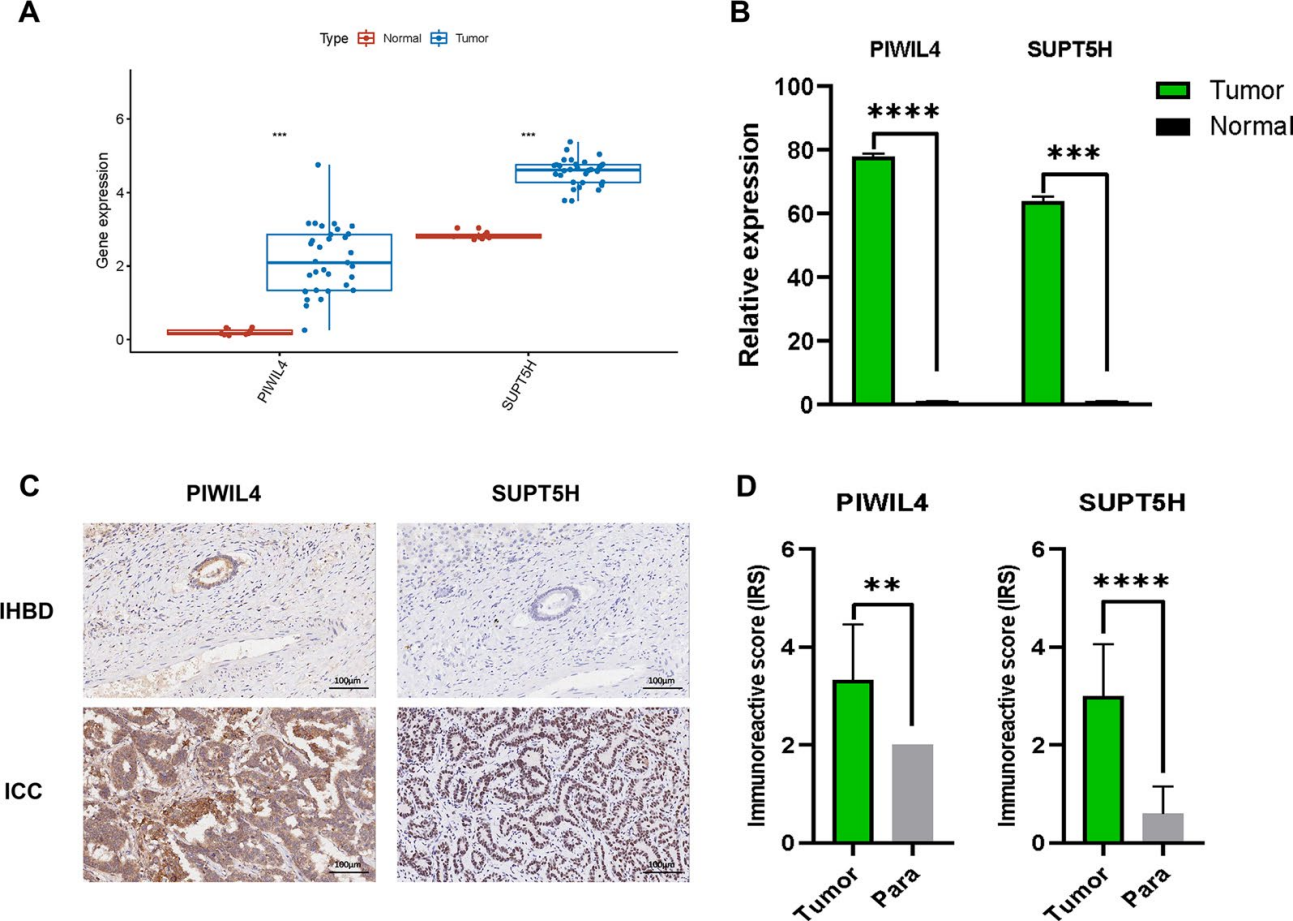
Validation of proteins expression. **A** The differentially-expressed level of PIWIL4 and SUPT5H were shown in boxplot. **B** The PIWIL4 and SUPT5H expression level was detected by qRT-PCR, h18S was used as internal control. **C** Representative IHC images of the PIWIL4 and SUPT5H expression in ICC and para-carcinoma tissues (200 × magnification). **D** Immunoreactive score (IRS) of the PIWIL4 and SUPT5H in ICC samples and normal tissues. (* P < 0.05; ** P < 0.01; *** P < 0.001, **** P < 0.0001)

We verified the proteins PIWIL4 and SUPT5H using qRT-PCR and IHC in ICC. First, we analysed the transcriptomic data and demonstrated significantly upregulated levels of the proteins in ICC tissues (Fig. 3A), and the qRT-PCR results showed similar expression trends (Fig. 3B). We further performed IHC to validate the expression of these proteins. The IHC results showed that PIWIL4 was located in the cytoplasm, and SUPT5H was located in the nucleus, while PIWIL4 and SUPT5H were both significantly upregulated in ICC (Fig. 3C, D).

### Development and validation of RBP-related prognostic signature

**Fig. 4:**
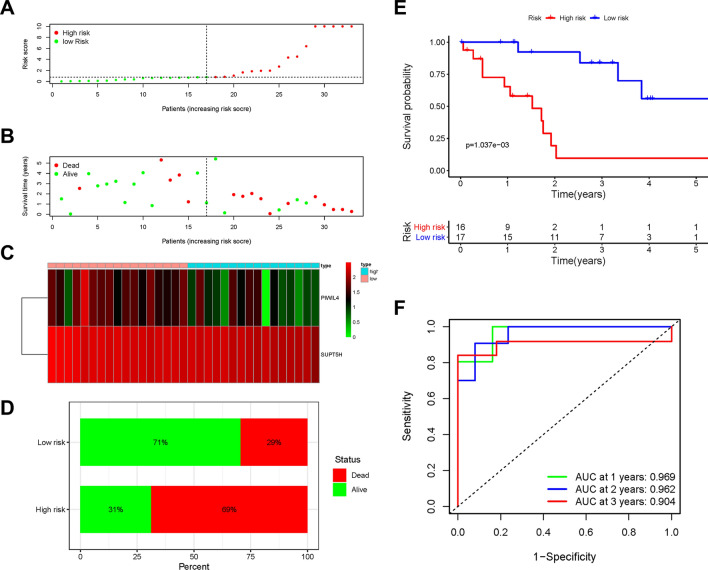
**A**–**D** Survival condition plots, heatmap, barplot in training cohort. **E** Kaplan-Meier survival curve in training cohort. **F** Time-dependent ROC curves used to predict OS at 1, 2, and 3 years in training cohort

An optimal prognostic signature was constructed. The riskScore was calculated as follows: $\mathrm{riskScore }= (-0.660) *\mathrm{ PIWIL}4 + (-4.867) *\mathrm{ SUPT}5\mathrm{H}$. All patients in the training and testing cohorts were allocated into high- and low-risk groups using their median riskScore. In the training cohort, survival condition plots and bar plots showed the mortality difference among the risk groups and revealed the differential expression patterns of DE-RBPs (Fig. 4A–D). Moreover, the KM survival curve indicated that the high-risk group had a poorer prognosis and a shorter OS than the low-risk group (p < 0.01, Fig. 4E). The AUC calculated at 1, 2, and 3 years to verify the model accuracy were 0.969, 0.962, and 0.904, respectively (Fig. 4F).

**Fig. 5:**
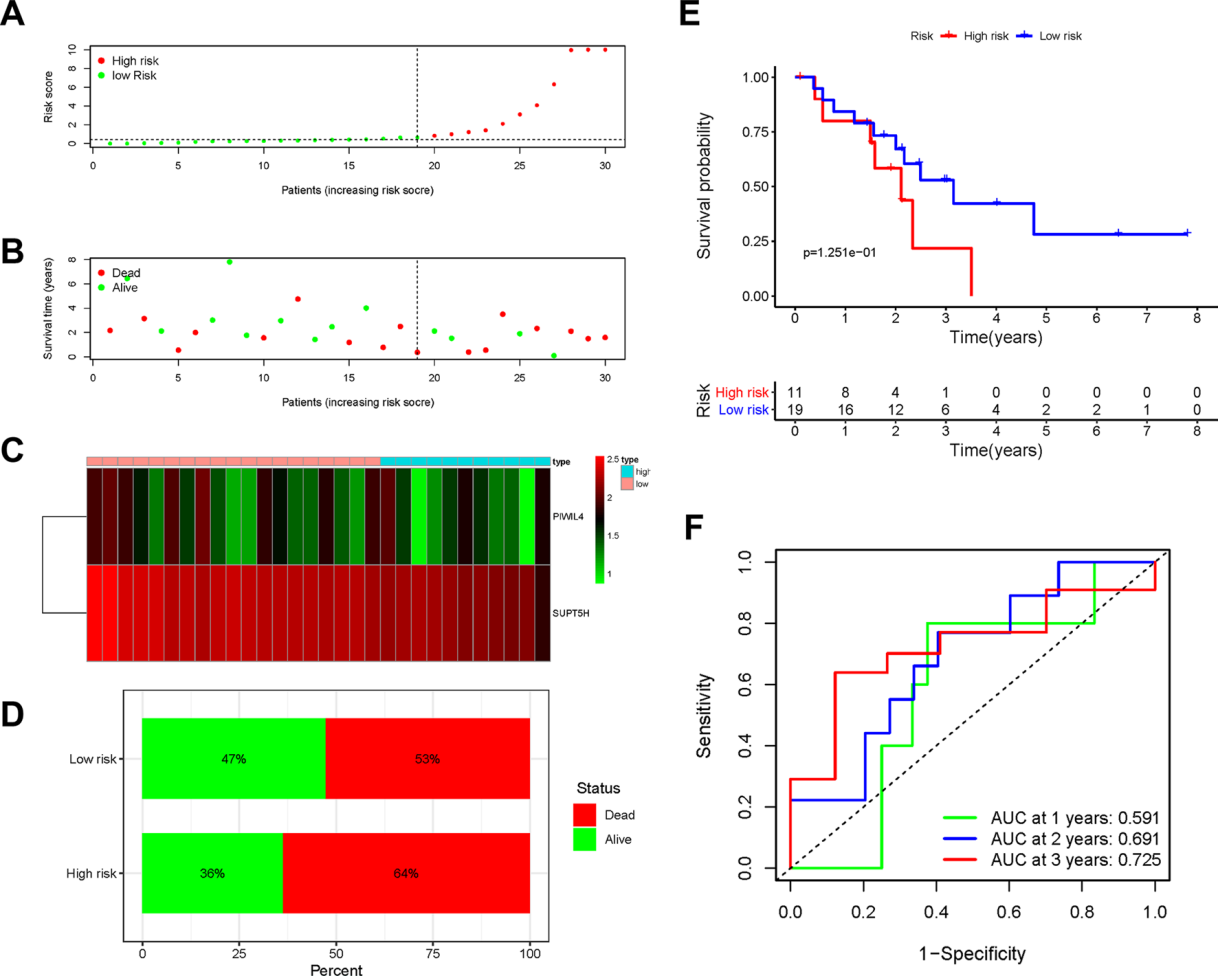
**A**–**D** Survival condition plots, heatmap, barplot in testing cohort. **E** Kaplan-Meier survival curve in testing cohort. **F** Time-dependent ROC curves used to predict OS at 1, 2, and 3 years in testing cohort

In the testing cohort, survival condition plots and bar plots showed a similar difference in that the high-risk group had higher mortality than the low-risk group (Fig. 5A–D). The KM survival curve showed a survival difference between the two groups even if p > 0.05 (Fig. 5E). The AUC at 1, 2, and 3 years were 0.591, 0.691, and 0.725, respectively (Fig. 5F). All the above results demonstrated that this signature could predict the prognosis of ICC patients with good predictive accuracy. In addition, these two RBPs were significantly correlated with one another in the training and testing cohorts (Additional file 2: Fig. S3).

### Gene set enrichment analysis

**Fig. 6:**
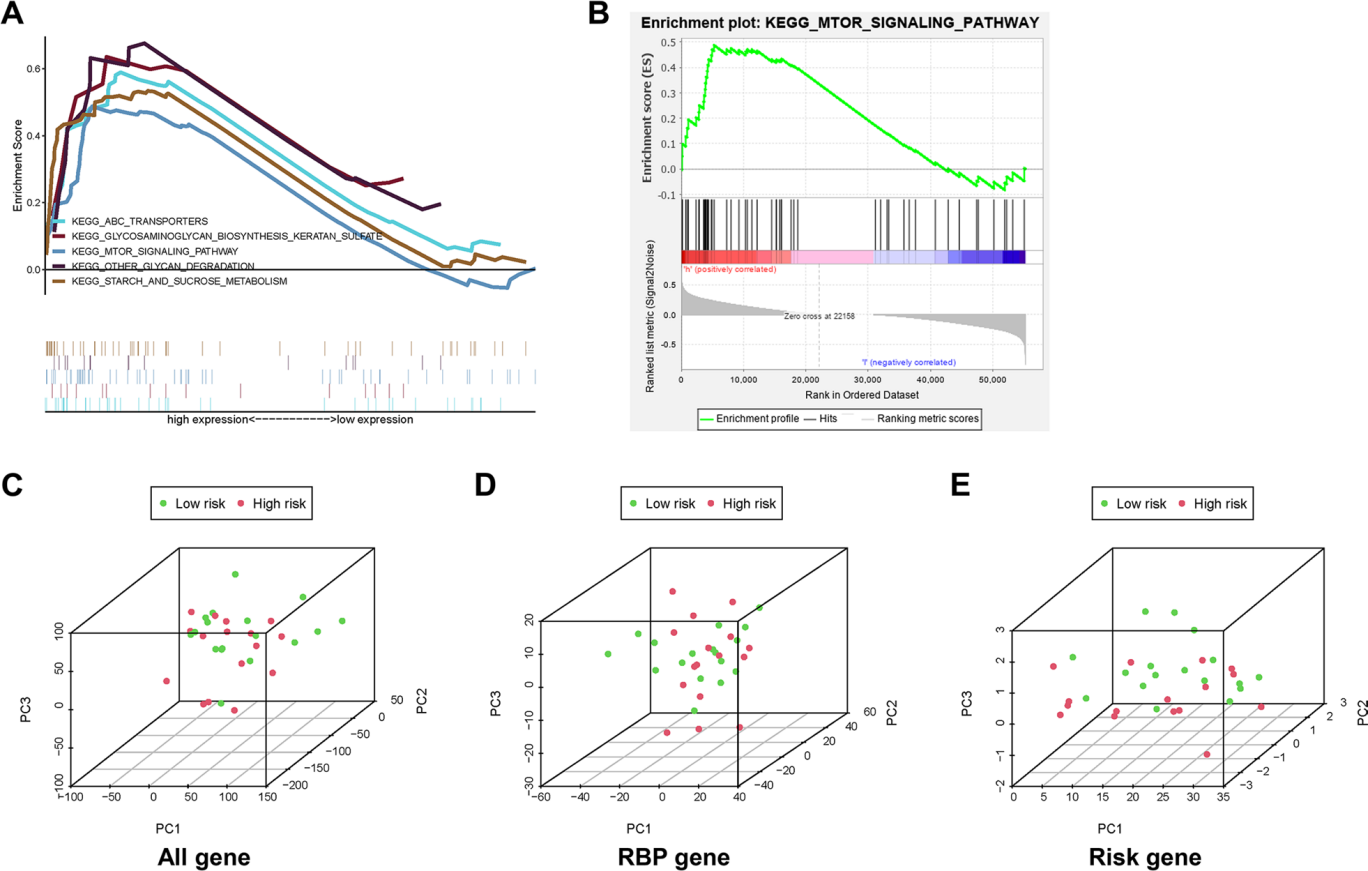
**A** Gene set enrichment analysis, **B** The significantly enriched mTOR signaling pathway, **C**–**E** Principal component analysis based on the whole genes, RBP-related genes, risk-related genes

The significant enrichment pathways included ABC transporters, glycosaminoglycan biosynthesis keratan sulfate, mTOR signalling pathway, other glycan degradation, and starch and sucrose metabolism (Fig. 6A, Additional file 2: Fig. S4, and Additional file 1: Table S7). The mTOR signalling pathway has been reported to be the key pathway in tumorigenesis and progression (Fig. 6B). The principal component analysis results showed that the model had a good risk classification (Fig. 6C–E).

### Immune cell infiltration and chemotherapeutics efficacy analysis

**Fig. 7:**
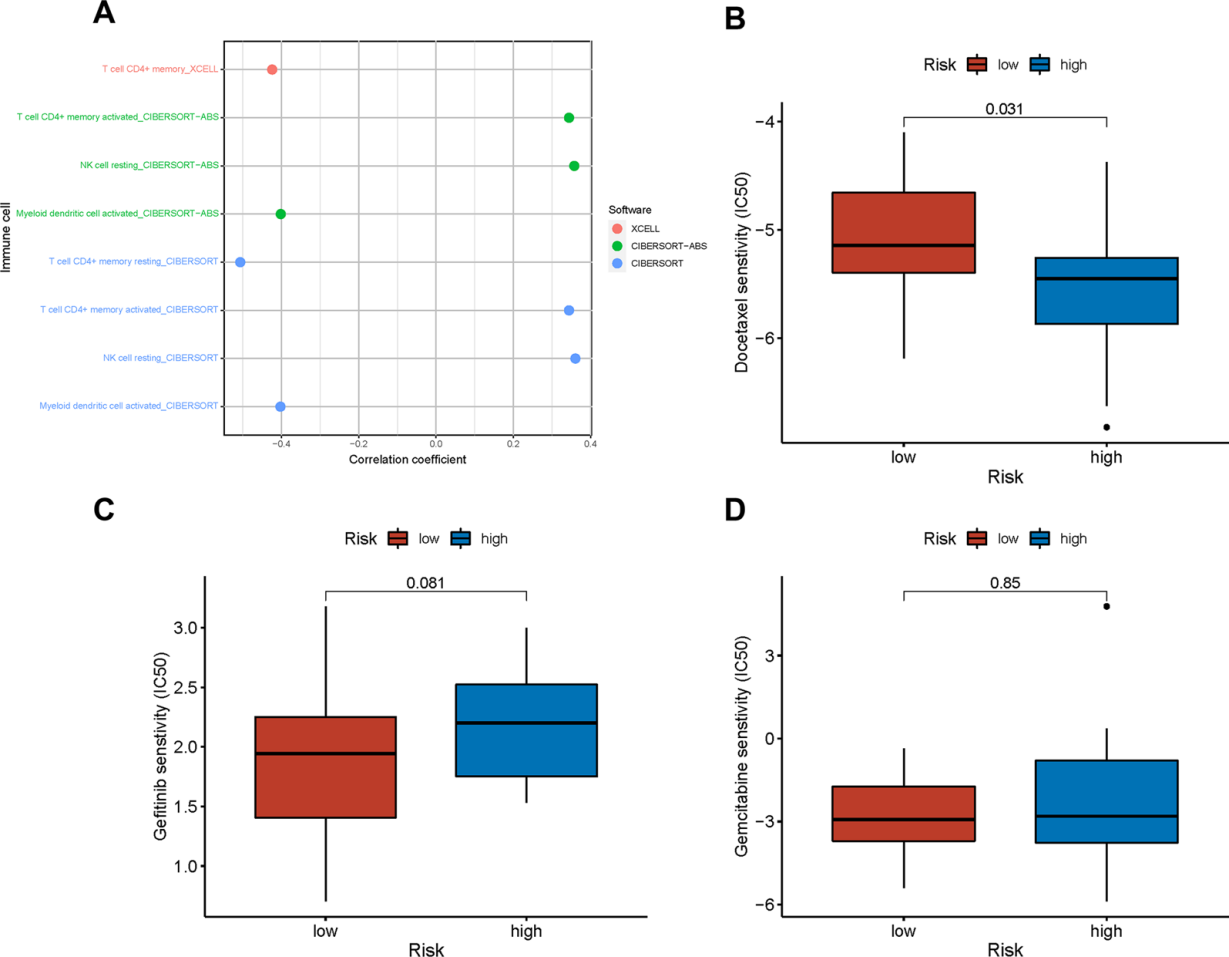
**A** Correlation analysis of signature with tumor-infiltrating immune cells. **B**–**D** The IC_50_ for frequently-used chemotherapeutics drugs, (**B**) Docetaxel, (**C**) Gefitinib, (**D**) Gemcitabine

We investigated the correlation of PIWIL4 and SUPT5H with the tumour microenvironment, and the results suggested that the high riskScore was positively related to the enrichment of resting natural killer (NK) cells and activated memory CD4 + T cells. Meanwhile, the decreased enrichment of memory CD4 + T cells activated myeloid dendritic cells and resting memory CD4 + T cells (Fig. 7A). Notably, the immune correlation of the two DE-RBPs is shown in Table 3, and PIWIL4 was demonstrated to be differentially expressed in groups with or without immunotherapy for melanoma (p = 0.0385)[41]. Subsequently, we aimed to investigate the relationship between the riskScore and common chemotherapeutic efficacy. The results showed that the low riskScore was positively correlated with the high IC_50_ of the chemotherapeutic agent docetaxel (p = 0.031) and low IC_50_ of gefitinib and gemcitabine (Fig. 7B–D); however the latter two agents were not statistically significant.

**Table 3:** Immune-related markers and mechanism analysis of PIWIL4 and SUPT5H

Gene	Types	Target	Spearman correlation analysis	p value	Survival	p value
PIWIL4	Chemokin	CCL24	− 0.434	p < 0.001		p = 0.00626
	receptor	CCR9	0.344	0.0408		
SUPT5H	Chemokin	CXCL5	− 0.419	0.0116		p = 0.0234
		CXCL12	0.405	0.0148		
		CXCL16	− 0.359	0.0321		
	Immunoinhibitor	IL10RB	0.44	0.00775		
		PVRL2	0.52	0.00133		
	Immunostimulator	CD276	0.357	0.0331		
		CXCL12	0.405	0.0148		
		ENTPD1	− 0.364	0.0297		
		TMEM173	− 0.334	0.0473		
		TNFSF4	− 0.337	0.0447		
		TNFSF13	− 0.382	0.0222		
	CHOL_MHC	HLA-DOB	− 0.367	0.0282		
	CHOL_TIL_TEM	Tem CD4 cells	− 0.349	0.0376		
	CHOL_TIL_Th2	Th2 cells				
	CHOL_TIL_Th17	Th17 cells	− 0.421	0.0112		

PIWIL4					
Cancer type	Drug	Group	Res vs N-Res	Log2(FC)	p value
Melanoma	Anti-PD-1(pembrolizumab and nivolumab)	All	14 vs 12	− 0.524	0.0385

*Res* The numbers of responders, *NRes* The numbers of non-responders

### Clinical relevance analysis and nomogram construction

**Fig. 8:**
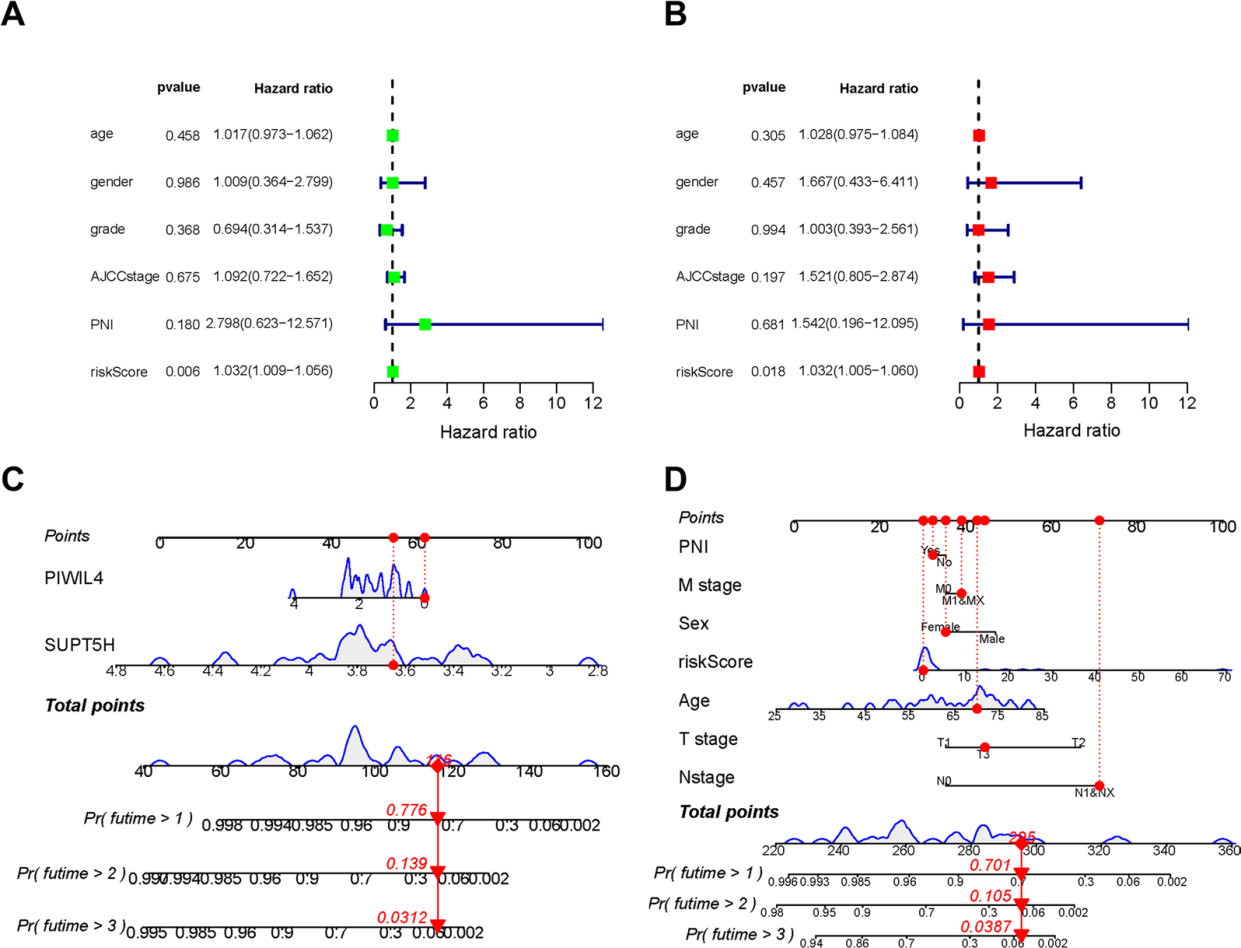
**A**–**B** Forest plot of univariate and multivariate regression analyses. **C**–**D** Two nomograms for predicting OS at 1, 2, and 3 years

To further reveal the clinical predictive power of PIWIL4 and SUPT5H, univariate and multivariate Cox regression analyses were performed based on all the clinicopathologic characteristics and riskScore. The results suggested that the riskScore had an independent predictive value (Fig. 8A, B, Additional file 1: Table S8). Further survival analyses stratified by clinical characteristics were carried out, which showed that high-risk patients had poor long-term survival in all clinical characteristics for stratification survival analyses (Additional file 2: Fig. S5).

Next, we integrated the transcriptomic data and clinicopathologic characteristics into two nomograms for predicting 1, 2, 3 years survival rates (Fig. 8C, D). The multivariable ROC curve showed that the riskScore had the best predictive accuracy (AUC = 0.993, Additional file 2: Fig. S6A), and the calibration curves showed a good agreement between predicted and actual 1, 2, and 3 year survival rates (Additional file 2: Fig. 6B–G). The clinical usefulness of the two nomograms was accurate and steady in predicting the long-term prognosis of patients with ICC.

## Discussion

Similar to most malignancies, the long-term survival and therapeutic effects of patients with ICC remain poor [4]. Recently, with the increasing development of immunotherapy and targeted therapy, exploring novel tumour biomarkers has become a promising field of study [6]. In the present study, two prognostic biomarkers, PIWIL4 and SUPT5H, and their corresponding immune-mediated status for ICC were identified and validated. PIWIL4 and SUPT5H were both significantly upregulated in ICC and had the potential to become prognostic biomarkers. We then developed a prognostic signature, which could allocate the patients into high- and low-risk groups to distinguish long-term survival. In addition, the potential mechanism of the signature was revealed via GSEA, which showed that the biomarkers are involved in the mTOR signalling pathway in tumour progression. Increasing evidence has also demonstrated that the mTOR signalling pathway plays an essential role in the initiation and progression of ICC, and could be considered as a future therapy target [42, 43]. The prognostic signature was a powerful predictor of ICC prognosis, with its predictive capability validated in the testing cohort. Notably, we constructed two nomograms based on different detection methods, which provided different predictive tools for different patients. All calibration curves also showed an agreement between the probability of predictive survival and actual survival in 1, 2, and 3-year. Furthermore, the multivariate ROC curve showed that the riskScore had the best predictive accuracy.

PIWIL4 is a member of the P-element-induced wimpy testis (PIWI) protein family that was first discovered in germline cells [44]. PIWIL proteins bind a unique type of non-coding small RNA called piRNAs (PIWI-interacting RNAs) to form the piRNA/piwi complex, which influences protein regulation and epigenetic regulation, etc. [45]. Recently, PIWI family proteins have been considered as prognostic markers for various malignancies. A systematic review and meta-analysis showed that PIWI family proteins have the potential to indicate the prognosis of various cancer, and lower PIWIL4 expression levels indicate worse prognosis in cancer [46]. Iliev et al. demonstrated that decreased expression levels of PIWIL4 indicated worse long-term survival in patients with renal cell carcinoma [47]. Li et al. also revealed that patients with low levels of PIWIL4 protein expression had a poor prognosis, and that PIWIL4 plays an important role in maintaining pancreatic cell homeostasis [48], which are results that are all consistent with our study. Notably, Mishra, N. K et al. study identified that PIWIL4 involve in DNA methylation and predict the prognosis, and can be used as a key prognostic biomarker in ICC [49]. In the present study, we explored the prognostic value of PIWIL4 based its RNA-binding protein mechanisms, and elucidated prognostic power of PIWIL4 from new perspective. thus, PIWIL4 should be considered and verified as an important biomarker in ICC. Recent evidence indicates that SUPT5H is a vital transcription promoter-binding protein involved in transcriptional elongation [50–52]. Lone et al. revealed a vital role of SUPT5H in regulating the expression levels of genes that control proliferation, migration, cell cycle, and apoptosis in breast cancer cases [53]. However, no study to the best of our knowledge has demonstrated the relationship between SUPT5H expression levels and the prognosis of patients with malignancies; our study being one of the first to identify SUPT5H as a prognostic biomarker in ICC, with low expression levels of SUPT5H reflecting poor prognosis, and it can be considered as a protective factor. This study provides novel research targets in tumour progression and prognosis of ICC.

To date, several prognostic signatures have been developed for predicting the survival of patients with ICC. In a previous study, Guo et al. comprehensively analysed and identified prognostic signatures, including seven mRNAs, for predicting recurrence in cholangiocarcinoma [54]. Mishra et al. identified nine genes which could also be strongly considered as prognostic markers of cholangiocarcinoma [49]. Huang et al. also identified three genes as pivotal tumour antigens of cholangiocarcinoma, which could benefit mRNA vaccine development [15]. Furthermore, our team and Xie et al. developed and validated a prognostic signature based on lncRNA-seq data [32, 55].

However, even though RBPs have been identified to have a crucial effects [20, 23, 56], a vital RBP-related risk model for ICC has not been reported yet. To our knowledge, we are the first to identify and construct the risk signature in ICC patients using two key RBPs. Notably, in the present study, this signature was verified in independent real-world cohorts.

In addition, the tumour immune microenvironment plays a pivotal role in the response to tumour immunotherapy [57]. Recently, a variety of studies has reported that immune cell infiltration and expression of immune checkpoints directly affect patient prognosis [58–60]. Therefore, we further investigated the relationship between immune cell infiltration and the risk signature. The results suggested that the high riskScore had more enrichments of resting NK cells and activated memory CD4 + T cells. We also revealed the relationship between the immune landscape and each RBP. Therefore, this signature was able to depict the immune landscape in ICC. This signature could also predict the efficacy of chemotherapeutic agents like docetaxel. Consequently, the signature could be considered as an indicator of ICC patients who may benefitted from immunotherapy and some chemotherapeutic drugs.

Although these findings and our model had a good capacity to predict the long-term survival of ICC patients, there are some limitations to this study. First, all transcriptomic data was obtained from a public database, and retrospective analysis was performed. Thus, selection bias was inevitable. Notably, we validated that PIWIL4 and SUPT5H expression pattern in ICC using the laboratory experiments, which indicated that both PIWIL4 and SUPT5H might involve tumour progression of ICC. Furthermore, we tentatively explored whether PIWIL4 and SUPT5H were highly related to the mTOR signalling pathway; however, the transcriptional regulation mechanism of PIWIL4 and SUPT5H still needs to be revealed, as well as their effect on tumour progression and long-term survival needs to be clarified. Thus, further laboratory experiments for validation of the mechanism are required.

## Conclusion

PIWIL4 and SUPT5H were identified and validated as novel prognostic biomarkers via bioinformatics and laboratory experiments, and a signature was developed to predict the prognosis and risk classifiers of ICC patients. We also identified the immune landscape of the two markers in the ICC immune microenvironment. This study offers a promising perspective for exploring biomarkers in ICC.

## Supplementary Information

**Additional file 1: Table S1.** Primary antibodies used for immunohistochemistry (IHC). **Table S2.** Identification of RNA-binding proteins. **Table S3. **Clinical information of all patients with intrahepatic cholangiocarcinoma. **Table S4.** Differentially expressed analysis of RBP. **Table S5.** Detailed information protein-protein interaction network. **Table S6. **Copy number variation data obtained from TCGA database. **Table S7.** Gene set enrichment analysis. **Table S8. **Univariate and multivariate Cox regression analysis for identifying idependently prognostic factors.

**Additional file 2: Figure S1.** Functional enrichment analysis of up and down RNA-binding proteins. **Figure S2.** **A** The protein-protein interaction (PPI) network; **B**–**D** subset visualization on Cytoscape. **Figure S3.** Spearman correlation analysis of PIWIL4 and SUPT5H in the TCGA and GEO databases. **Figure S4.** Gene set enrichment analysis. **Figure S5.** Kaplan-Meier survival analysis of the signature stratified by clinical characteristics. **Figure S6.** A Multivariable AUC values from ROC.

## Data Availability

The public data used to support the results of this study can be obtained from The Cancer Genome Atlas (TCGA) (https://cancergenome.nih.gov/), GEO database (https://www.ncbi.nlm.nih.gov/geo/), UCSC Xena (http://xena.ucsc.edu/) and TISIDB database (http://cis.hku.hk/TISIDB/).

## Abbreviations

ICC: Intrahepatic cholangiocarcinoma
RBPs: RNA binding proteins
TCGA: The Cancer Genome Atlas
GEO: Gene Expression Omnibus
AUC: Area under the curve
CNV: Copy number variation
Ct: Cycle threshold
FC: Fold change
GTF: Gene transfer format
GO: Gene ontology
GSEA: Gene set enrichment analysis
IHC: Immunohistochemistry
IRS: Immunoreactive score
ICI: Immune cell infiltration
KEGG: Kyoto Encyclopaedia of Genes and Genomes
MSI1: Musashi-1
MSI2: Musashi-2
OS: Overall survival
PCA: Principal component analysis
PNI: Peripheral nerve infiltration
ROC: Receiver operating characteristic
DE-RBPs: Differentially expressed RNA binding proteins
KM: Kaplan–Meier
qRT-PCR: Quantitative real-time PCR
NK: Natural killer cell
IC_50_: Half inhibitory concentration
PPI: Protein–protein interaction
