# Supplementary material for: PIWIL4 and SUPT5H combine to predict prognosis and immune landscape in intrahepatic cholangiocarcinoma
Source: Cancer Cell Int. 2021 Dec 7;21:657. doi: 10.1186/s12935-021-02310-2 (PMC8649993; doi:10.1186/s12935-021-02310-2)
Supplement: Supplementary file 2 — Additional file 2: Figure S1. Functional enrichment analysis of up and down RNA-binding proteins. Figure S2. A The protein-protein interaction (PPI) network; B–D subset visualization on Cytoscape. Figure S3. Spearman correlation analysis of PIWIL4 and SUPT5H in the TCGA and GEO databases. Figure S4. Gene set enrichment analysis. Figure S5. Kaplan-Meier survival analysis of the signature stratified by clinical characteristics. Figure S6. A Multivariable AUC values from ROC. [file 12935_2021_2310_MOESM2_ESM.docx]

Additional file 1

PIWIL4 and SUPT5H combine to predict prognosis and immune landscape in intrahepatic cholangiocarcinoma

**Running Title:**

Novel biomarkers in intrahepatic cholangiocarcinoma

Additional file 1: **FigureS1**


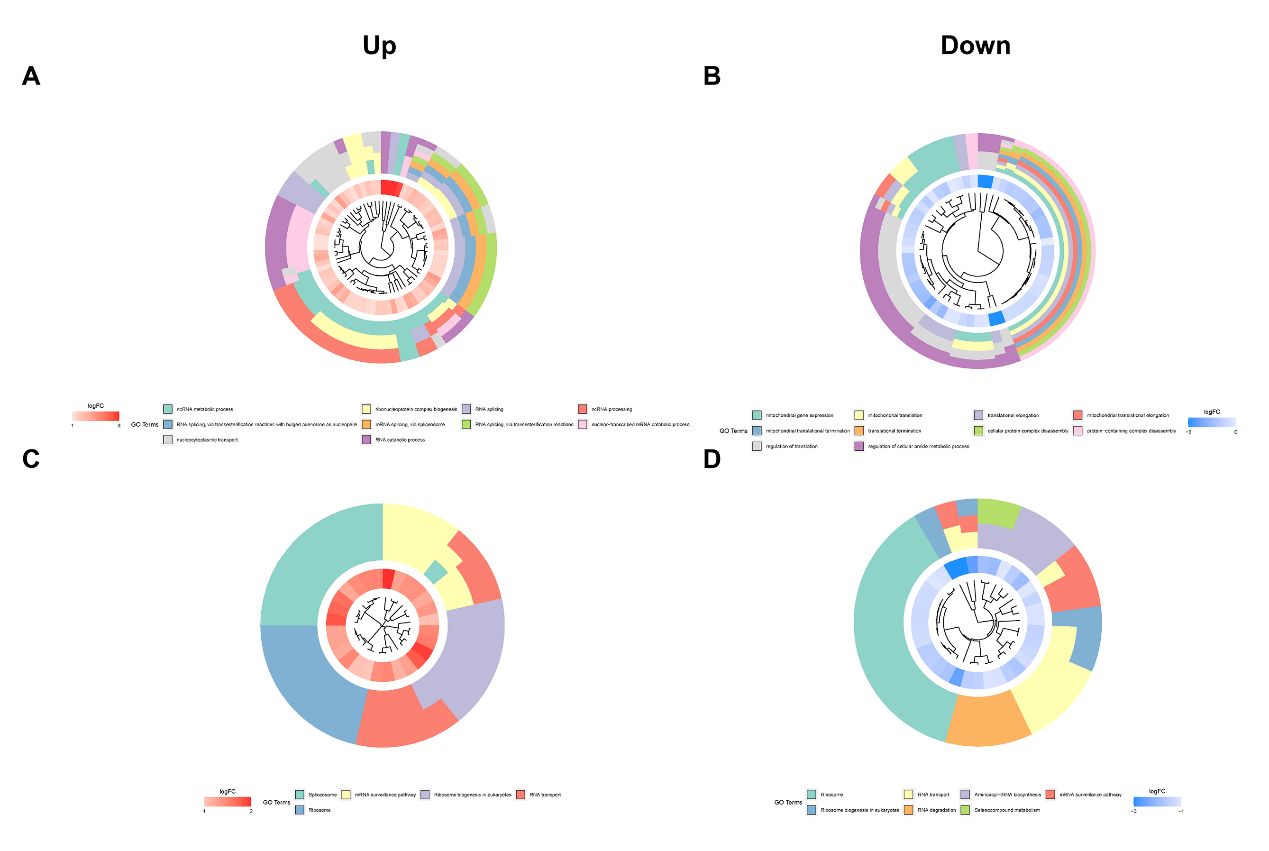


Additional file 1: Figure S1: Functional enrichment analysis of up and down RNA-binding proteins. A-B GO enrichment analysis; C-D KEGG pathway enrichment analysis.

Additional file 1: **FigureS2**


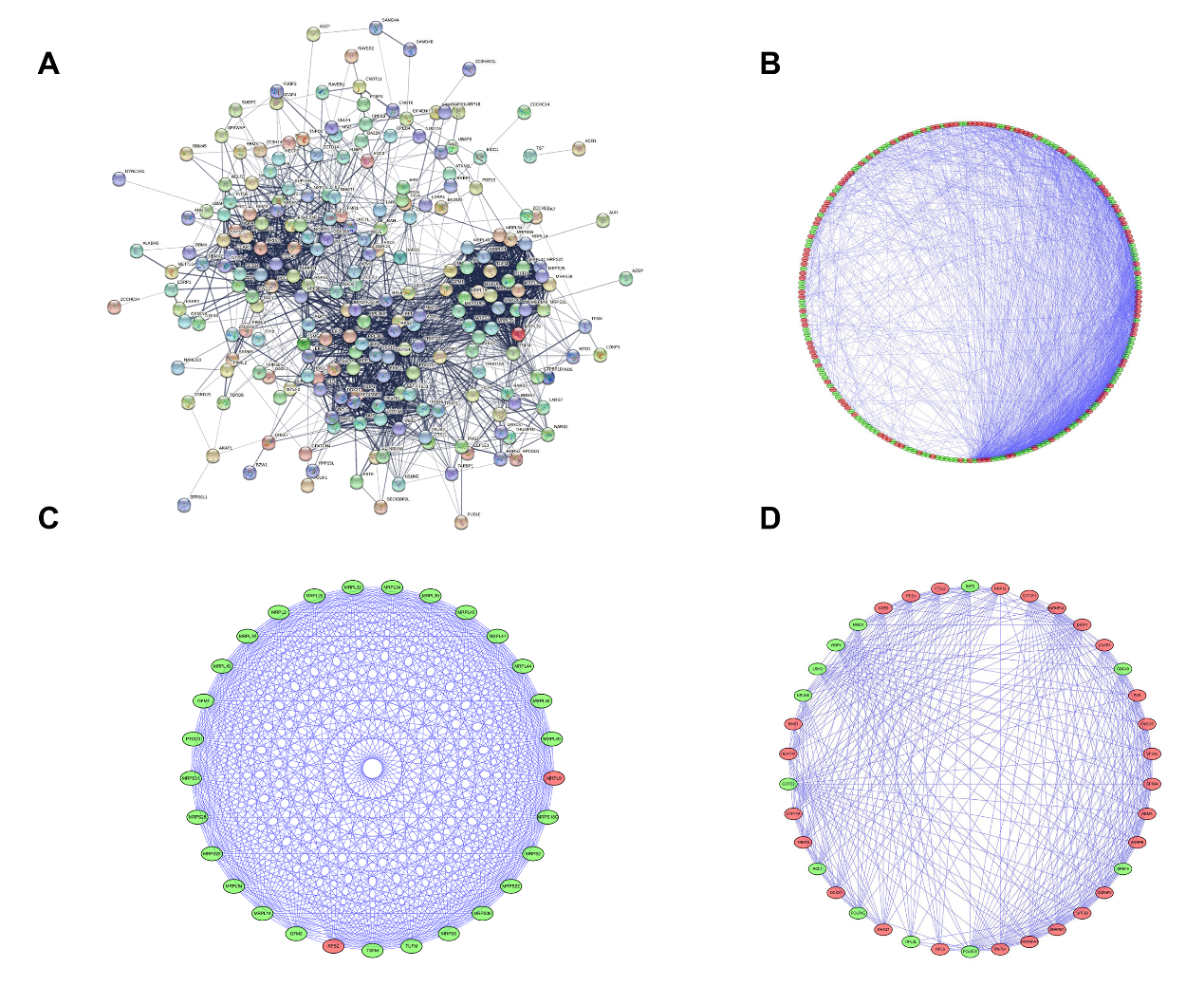


Additional file 1: Figure S2: A The protein-protein interaction (PPI) network; B-D subset visualization on Cytoscape.

Additional file 1: **FigureS3**


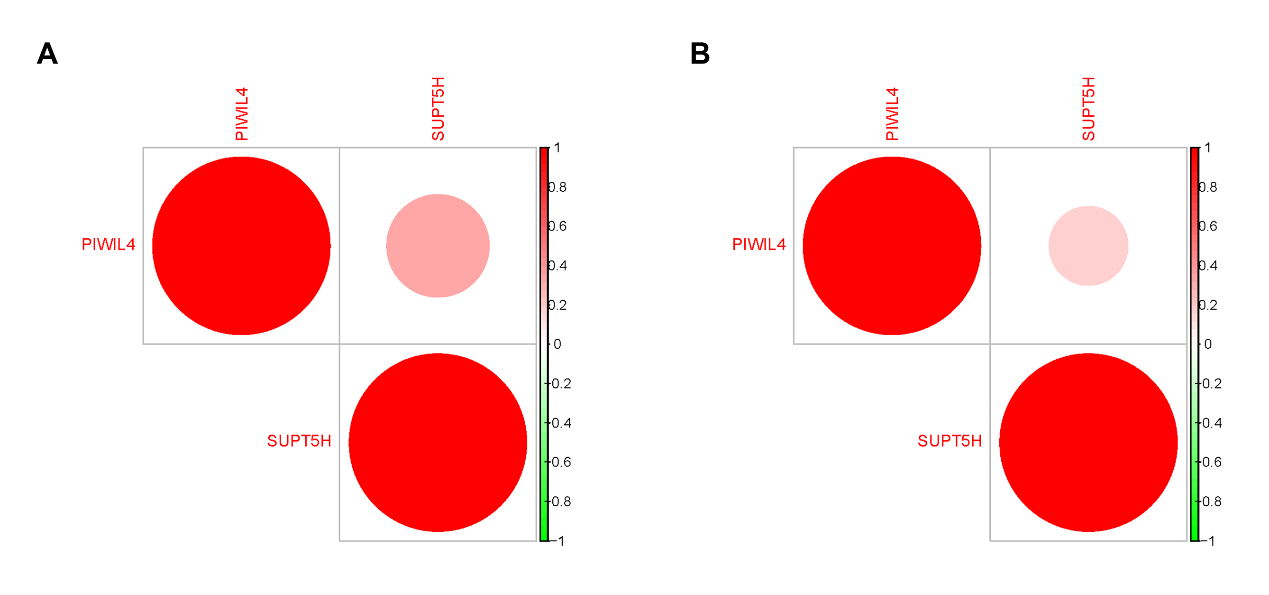


Additional file 1: Figure S3: Spearman correlation analysis of PIWIL4 and SUPT5H in the TCGA and GEO databases.

Additional file 1: **FigureS4**


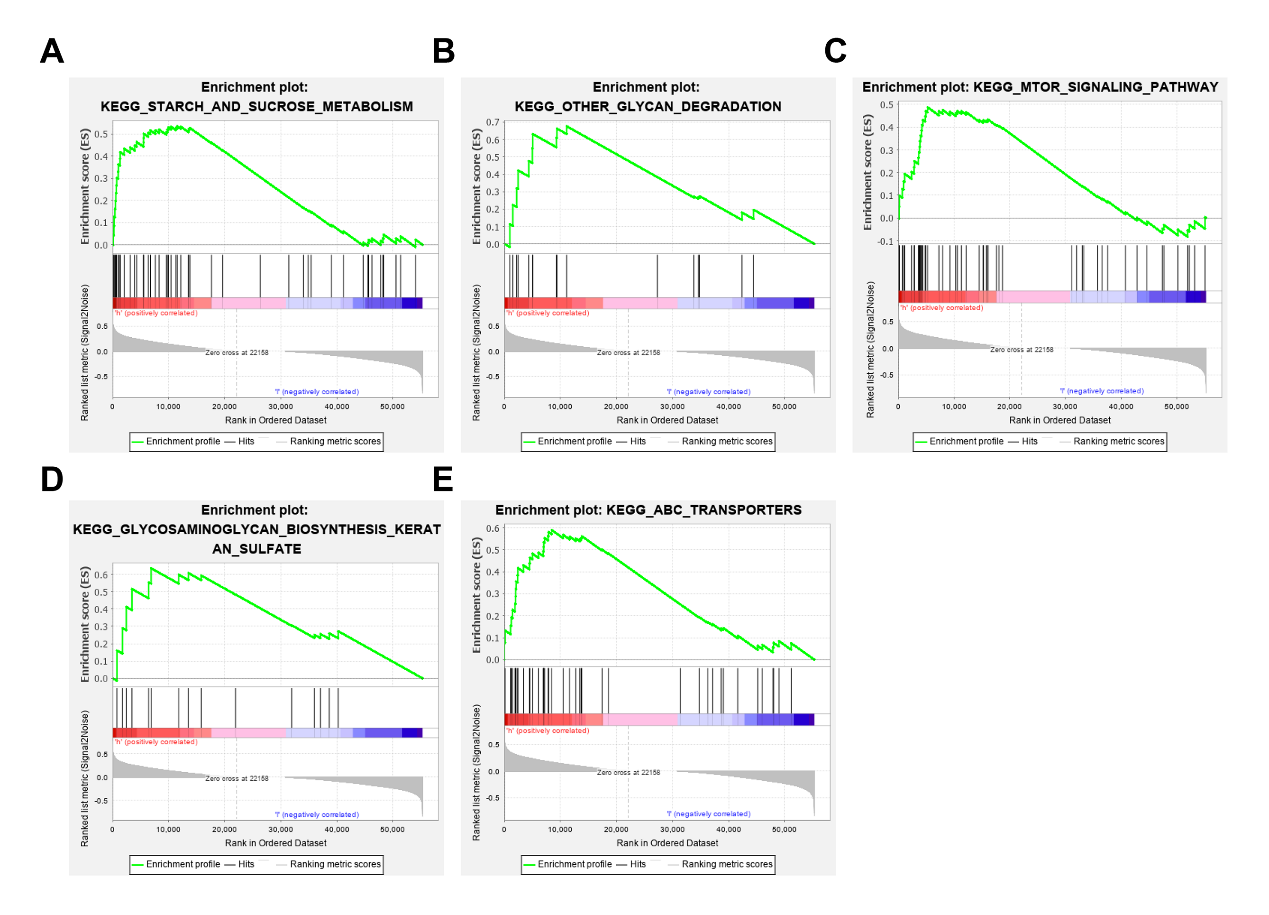


Additional file 1: Figure S4: Gene set enrichment analysis. A-E Top five significantly enriched KEGG pathways.

Additional file 1: **FigureS5**


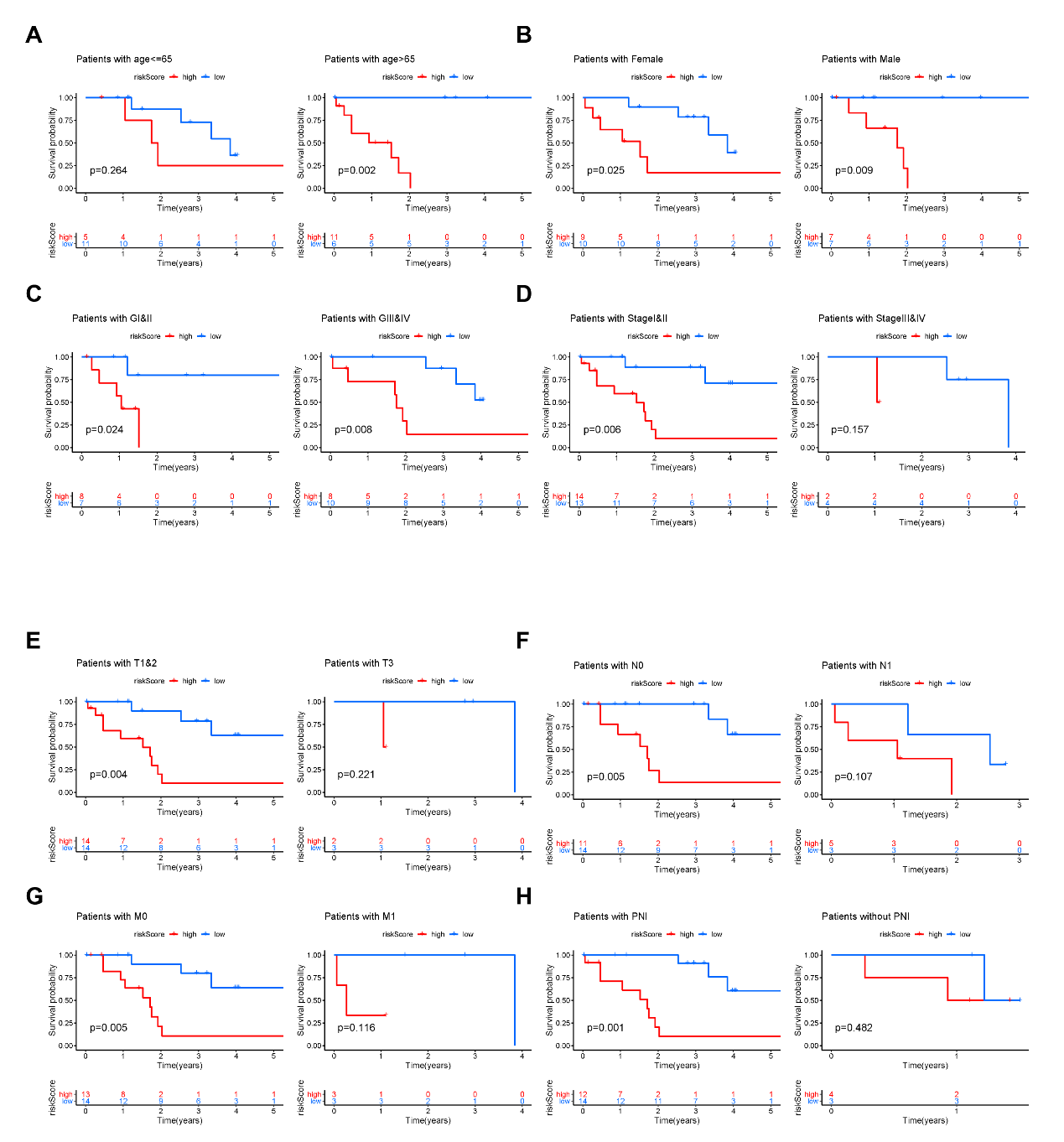


Additional file 1: Figure S5: Kaplan-Meier survival analysis of the signature stratified by clinical characteristics. A age; B sex; C grade classification; D AJCC stage; E T stage; F N stage; G M stage; H PNI.

Additional file 1: **FigureS6**


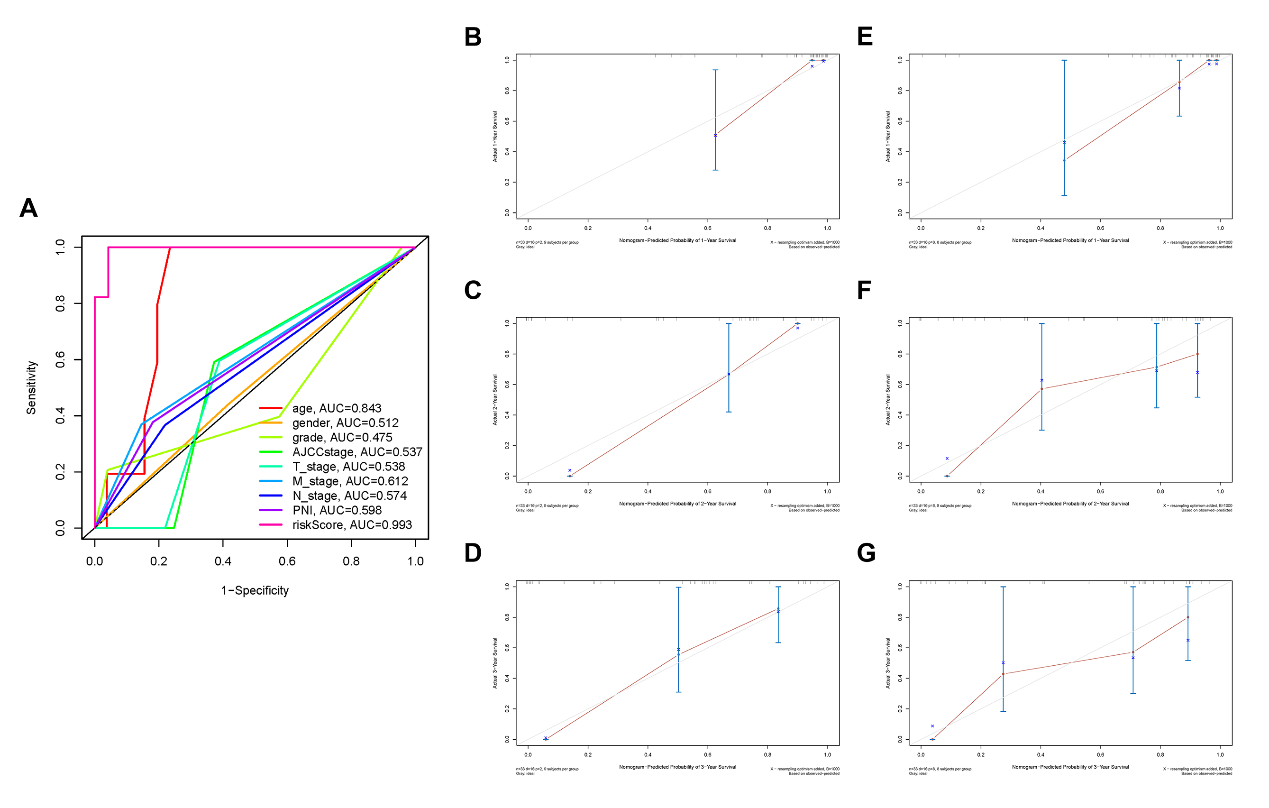


Additional file 1: Figure S6: A Multivariable AUC values from ROC. B-G Calibration curves showing the probability of 1-, 2-, and 3-year OS between the two nomograms prediction and practical observation. (B-D nomogram1; E-G nomogram2)
